# From QTL to QTN: Candidate Gene Set Approach and a Case Study in Porcine IGF1-FoxO Pathway

**DOI:** 10.1371/journal.pone.0053452

**Published:** 2013-01-14

**Authors:** Minghui Wang, Qishan Wang, Yuchun Pan

**Affiliations:** 1 School of Agriculture and Biology, Shanghai Jiao Tong University, Shanghai, People’s Republic of China; 2 Shanghai Key Lab of Animal Biotechnology, Shanghai, People’s Republic of China; National Cancer Center, Japan

## Abstract

Unraveling the genetic background of economic traits is a major goal in modern animal genetics and breeding. Both candidate gene analysis and QTL mapping have previously been used for identifying genes and chromosome regions related to studied traits. However, most of these studies may be limited in their ability to fully consider how multiple genetic factors may influence a particular phenotype of interest. If possible, taking advantage of the combined effect of multiple genetic factors is expected to be more powerful than analyzing single sites, as the joint action of multiple loci within a gene or across multiple genes acting in the same gene set will likely have a greater influence on phenotypic variation. Thus, we proposed a pipeline of gene set analysis that utilized information from multiple loci to improve statistical power. We assessed the performance of this approach by both simulated and a real IGF1-FoxO pathway data set. The results showed that our new method can identify the association between genetic variation and phenotypic variation with higher statistical power and unravel the mechanisms of complex traits in a point of gene set. Additionally, the proposed pipeline is flexible to be extended to model complex genetic structures that include the interactions between different gene sets and between gene sets and environments.

## Introduction

The function of any biological process is determined by both its composition and its structure. The genetic architecture that links quantitative traits operates under the same conditions, wherein both gene identities as well as relationships amongst genes determine the phenotype. Over the past two decades, the candidate gene approach and linkage disequilibrium analysis have been used to discover QTGs (Quantitative trait genes) and QTNs (*Quantitative* Trait Nucleotide). The candidate gene approach is based on a prior knowledge including the physiological mechanisms described in other studies [1], for example, the estrogen receptor (*ESR)* gene [2]. Linkage disequilibrium analysis based on well-characterized populations has driven QTL (quantitative trait locus, a fragment of DNA covering one or several genes) mapping nearly to completion for almost all major livestock species. This approach exploits crosses of reference strains or individuals related through a known pedigree. Up to now, a huge amount of information has been accumulated and deposited in Animal QTLdb(http://www.animalgenome.org/QTLdb/ [3]), a valuable resource which is freely available for further mining.

In order to make use of the previous QTL mapping results, we proposed a complementary, alternative approach, named candidate gene set approach (CGSA), to identify QTG and QTN in a specific set of related genes such as regulatory networks, pathways, etc. The CGSA was designed to decrease false discoveries by considering a functional set and thus improve statistical power. The performance of the proposed approach was assessed by simulation based on 820 hydrid pigs and demonstrated by a real example of growth traits of 111 hydrid pigs.

## Materials and Methods

### 1 Candidate Gene Set Approach

#### 1.1 Defining candidate gene set

Gene set, as the name suggests, is a group of genes, which are functionally or structurally related to each other. There are many possible sources used for defining gene set, including gene ontology annotations [4], KEGG pathways [5], Molecular Signatures Database (MSigDB) [6], co-expression genes from microarray analysis, etc. They may even be chromosome regions covered by related QTL. The sources above are not an exhaustive list, but give a good idea on the types of analyses that can be done using the methods we described in this study. Parts of the gene sets can be selected as candidate gene sets based on some prior information for further analyses.

For animals (livestock) with a poor annotation of genes, we can define candidate gene set by comparative genomics, namely identifying orthologous genes in livestock by Blast searching, because researches on model animals have produced a huge amount of information. When such search indicates a livestock gene with unknown mapping data, the genes adjacent to the target gene in the query, can be similarly searched in the livestock genome in order to infer the location of livestock gene based on synteny. Recently, our group have developed a functional annotation platform called SNPpath for animals (livestock) SNP [7], and it can provide functional annotations of livestock SNP markers from several commercial platforms and dbSNP database. The web server may be particularly beneficial for the analysis of combining SNP association analysis with the CGSA.

#### 1.2 Mapping genes to QTL

As mentioned above, by fine design of experiments, QTL mapping has obtained a huge amount of information, including QTL location (chromosome, location, and location span), flanking markers, peak markers, test statistics, QTL effects and traits. For example, up to June of 2012, there were 6,818 QTLs that represent 585 different traits for pig [3]. If a gene within the candidate gene set can be mapped to the confidence interval of QTL that have some physiological relevance to the trait, it will be reasonably considered as primary candidate QTGs [8], especially, overlapping with multiple QTL of interest. Some QTL in the pig QTLdb have very large confidence interval, so we suggest that research should set a limitation of confidence interval by considering overlapping with multiple QTL of interest. We have successfully written a Perl script to relatively locate genes within the interval of the QTL.

#### 1.3 Predicting potential SNPs

Though a few thousand of SNPs in animal are currently available, which comes from commercial array or annotation database like dbSNP, the SNP density in animal is much lower than that in human. The low SNP density might limit the power of identifying genes involved in the traits of interest. The ongoing genome sequencing project and the releasing of the genome draft assembly of animal have provided the opportunity to compare and detect SNP candidates using alignment algorithms [9]. Kerstens (2009) successfully used this strategy to mine novel SNPs of pig using whole genome shotgun dataset generated by the Danish-Chinese Pig Genome Sequencing Initiative [10]. Therefore, we could perform similar pipeline to identify candidate SNPs.

#### 1.4 SNP genotyping

The predicted candidate SNPs as well as SNPs from commercial array and annotation database need to be genotyped in a panel of target population for association studies. Owing to advances in the area of molecular genetics and improvements in genotyping technology, a large number of SNP genotyping techniques were used to type allele-specific products for SNPs of interest followed by genotype determination. In addition to high cost in genotyping and stringent requirements for accuracy, the throughput has been a critical factor in many farm animals studying with large sample populations. Therefore, in most cases, we need to make a reasonable compromise between throughput and cost. For instance, typing SNP candidates overlapped with QTLs or that in all members of gene set is determined by cost to some extent.

#### 1.5 Association test

The CGSA method makes an attempt to answer the following questions: First, whether is there the association between genetic variation and phenotypic variation in the gene set level? Second, if the gene set is significantly associated with some traits, which SNPs or combinations play a key role?

To answer the two questions, we first used the following Mixed Liner Model (MLM) to perform our CGSA on each measured trait.

The matrix format of this model is that:
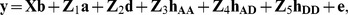
where **b** is a vector of fixed effects, **a** is vector of random additive genetic effects of candidate gene set, **d** is vector of random dominance effect of candidate gene set, and 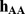, 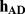 and 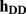 represent the interaction effects of additive-by-additive (AA), additive-by-dominance (AD) and dominance-by-dominance (DD) respectively. 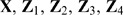 and 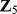 are the corresponding design matrix.

It is assumed that.
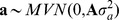


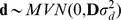


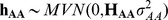


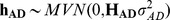


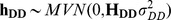


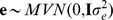



*MVN* represents multivariate normal distribution, **A** and **D** represent the corresponding additive (dominance) effect relationship matrix in candidate gene set level, respectively. The matrices can be estimated by the SNPs in select candidate gene set using the software of PLINK [11]. The PLINK estimates the probability of sharing 0, 1, or 2 alleles IBD (Identiy by Descent) for two subjects. The elements of A matrix can be expressed as 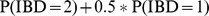 and the elements of D matrix can be calculated as 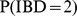 [11]. The corresponding epistatic variation effect relationship matrix could obtain by the Hadamard products of the A and D matrices [12]. The likelihood ratio test could be used to test the significance of the additive effects, dominance effect and epistatic effect for the candidate gene set.

Second, if the candidate gene set is significantly associated with some traits, the single SNP general linear model or multiple higher order interaction models such as MB-MDR method can be further used to study the genetic mechanism. MB-MDR method proposed by Calle et al. [13] not only assesses joint significance of multiple higher order interaction models at once, but also facilitates distinguishing between epistatic effects and contributing main effects to the multilocus signal via the ‘MB’ part in MB-MDR [14].

### 2 Simulations

#### 2.1 Simulation data

We compared the statistical power between our CGSA and the traditional candidate gene approach across a variety of heritabilities in a real experimental genotype data and simulated phenotype data produced by R program. The pig dataset was collected from 820 commercial female pigs. There were 51,385 SNPs scored on these individuals [15]. SNP markers were aligned to the corresponding Ensemble gene IDs (the Sus scrofa Build 9.2 downloaded from UCSC genome browser). The genes including 50 kb flanking regions (upstream and downstream of gene), were used for the assignment of SNPs to genes. The 57 SNPs mapped to 17 genes in the insulin and insulin growth factor-1/FoxO (IGF1-FoxO) signal pathway were used in the simulation procedure. All the makers with their positions are shown in Table S1.

#### 2.2 Simulation procedure

Two simulation experiments were conducted to assess the performance of our method. In the first simulation experiment, only additive genetic effects were simulated. Fifteen SNPs was randomly sampled from the IGF1/FoxO signal pathway as causal QTNs for the simulated traits. The distribution of these QTN effects was assumed as a normal distribution with mean of 0 and variance of 1. Phenotypes were calculated as the sum of additive effect and the random residual effect. The total additive effect was the sum of additive effects across all QTNs and the residual variance was computed as 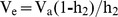, where 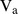 is the empirical additive genetic variance and h_2_ is the heritability. A residual error was assumed as a normal distribution with mean of 0 and variance of 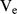. Five levels of heritability were analyzed (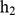 = 0, 0.05, 0.1, 0.25 and 0.5) in this simulation experiment.

In the second simulation experiment, additive effects, dominance effects and epistatic effects were simulated simultaneous. Thirteen SNPs from the IGF1/FoxO signal transduction pathway were assumed as causal QTNs for the simulated traits. All the main *effects* and the *effects* of interaction (*epistatic effects*) of the makers with their positions are shown in Table S2. Three paired SNPs were simulated as interacting QTN effect. It is assumed that the effects of paired SNPs were seen only if an individual is heterozygous at both QTNs. The simulated additive genetic variance and total genetic variance for the true 820 commercial female pigs were 6.05 and 9.01 respectively. The residual variance was set as 10. The power was calculated as the proportion of QTNs detected or the significant pathway with Type I error rate 0.01. A total of 1000 replications were conducted for each method. The R program written by the authors based on GAPIT software was used to perform analysis for the two simulation experiments [16].

### 3 Analysis of Real Data

#### Ethics statements

Prior approval by the Institutional Animal Care and Use Committee of Shanghai Jiao Tong University (Contract 2009-0068) was given for all experimental procedures involving animals in the present study.

#### Experimental design and procedure

The 111 Duroc ×Landrace ×Yorkshire (DLY) hybrid pigs were phenotyped on daily feed intake and body weight. All pigs were fed the same corn-soybeanmill based ration during the feeding period. Pigs were automatically weighed on their way to feeding using OSBORNE FIRE system. Feed intake and body weight (BW) were measured several times a day, which were then arithmetically averaged to one value for further analysis. If some measurements were missing, the remaining measurements were used and treated as a daily measurement. For generating variables representing BW changes from born to 100 kg and further analysis of the BW data, individual measurements were first smoothed using S-shaped logistic curves. In this study, we mainly focus on the corresponding timing intervals from birth to 30 kg (BW 30), from 30 to 60 kg (BW 60), from 60 to 100 kg (BW 100), average daily gain (ADG) and total feed intake (DFI). The heritability of BW 30, BW 60, BW 100, ADG and DFI are 0.39, 0.35, 0.34, 0.37 and 0.15 separately in present population.

#### Defining candidate gene set

To make use of the results from the related experiments of model organisms, we defined the gene sets based on our previous work, which presents the transcriptomic profile during skeletal muscle regeneration using time-course expression data and motif scanning information [17]. Our study recovered some signaling pathways involved in the regulation of muscle differentiation, and the IGF1/FoxO signal transduction pathway was selected to define a gene set for the current study.

#### Predicting potential SNP positions and SNP genotyping

Two data sources were used to predict SNP positions, one from the commercial array annotations (Illumina PorcineSNP60 BeadChip), and the other from the NCBI Trace repository. The identification of novel SNPs was performed by a 2-step process for the NCBI Trace repository. First, the shotgun sequences were assigned to a reference sequence (member form gene set) by clustering based on their sequence similarity. Secondly, the sequences within cluster were aligned to search for potential SNPs. A total of 111 genomic DNA samples from DLY hybrid pigs comprising 58 females and 53 castrates, were genotyped by SNaPshot method. The primer sequences for analysis of 23 identified SNPs are listed in **Table S3**.

#### Association test

The mixed linear model is as following, 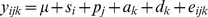, where 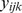 is the phenotypic value of each trait, 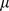
is the overall mean for each trait, 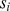 is the fixed gender effect (*i* = 1,2), 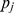
is fixed sire effect (j = 1,2,3), 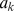 and 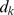 are the pathway additive effect and dominance effect of the k*th* individual,
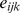
is the random residual effect. The additive and dominance genetic relationship matrix was estimated based on the TagSNPs in select candidate signaling pathway using the software of PLINK [11]. Then ASReml was used to estimate the pathway effects and test the significance level by the likelihood ratio test [18]. In this case study, we can take full advantage of the opportunities provided by MB-MDR model to study simultaneously the association of a group of genetic variants in predefined gene set, which can help us to holistically unravel the complex genetic structure of the studied trait and to gain insight into the biological processes and mechanisms.

## Results

### Simulation Results

**Figure 1:**
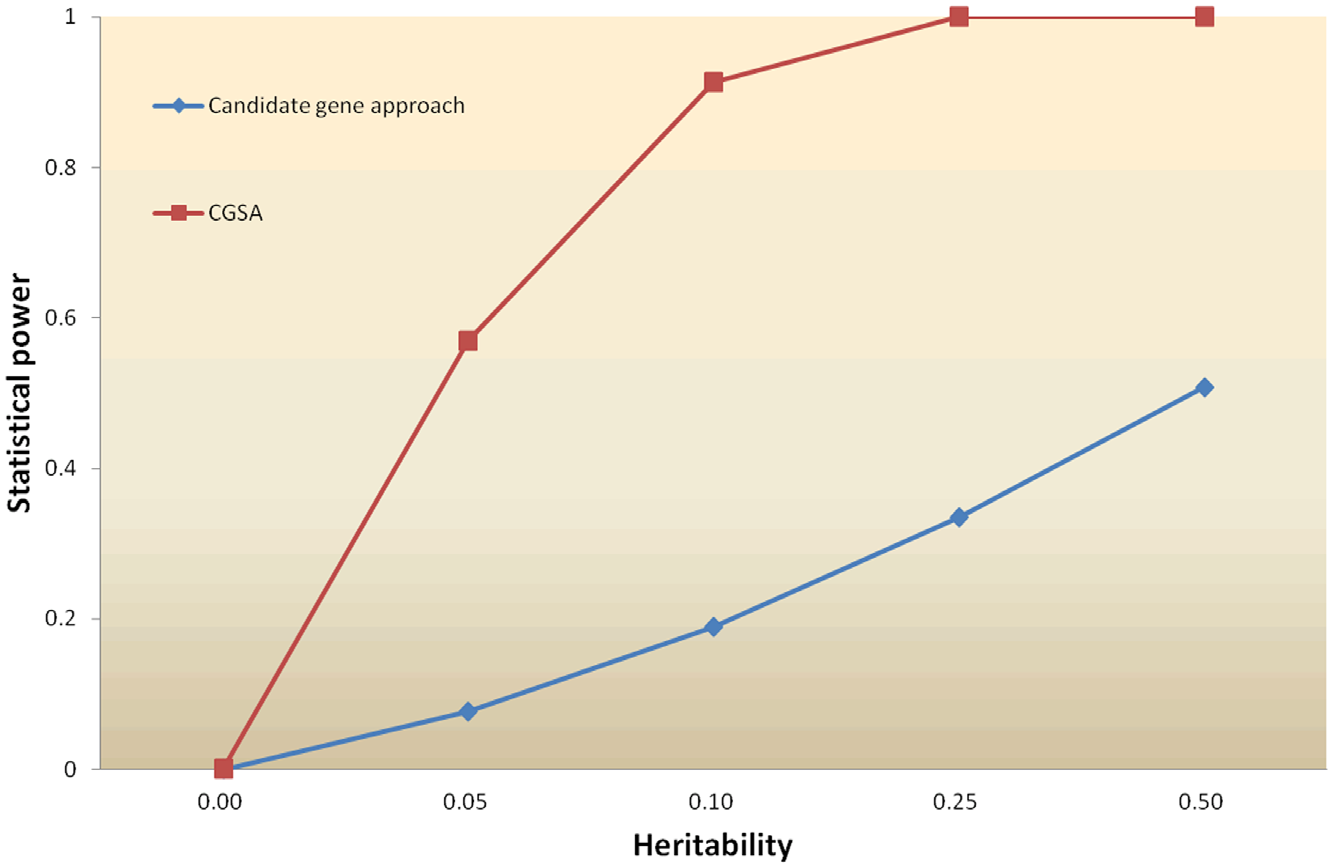
Statistical powers between CGSA and candidate gene approach under different heritability levels. The power was examined on a trait simulated from 15 causative mutations (QTNs). A total of 1000 replications were conducted for each method. The heritability of the trait varied from 0 to 0.5. The differences between our CGSA and candidate gene set approach with mid-level and low-level of heritability were greater than the one with high heritability.

For the first simulation experiment, generally, along all the levels of heritability investigated, as heritability increases, the statistical power of both methods increased as expected (Figure 1). Our CGSA performed higher statistical power over the traditional candidate gene approach across all kinds of heritabilities. The differences among these methods varied along the level of heritabilities. Figure 1 showed that the differences among the methods with mid-level and low-level of heritability were greater than the one with high heritability. For the second experiment, the statistical power of our CGSA and candidate gene approach was shown in **Table S4**. Compared to the traditional candidate gene approach, the CGSA performed the significantly higher statistical power for both additive effect and dominance effects and similar statistical power for epistatic effect in the present simulation scenario.

### Real Data Results

**Figure 2:**
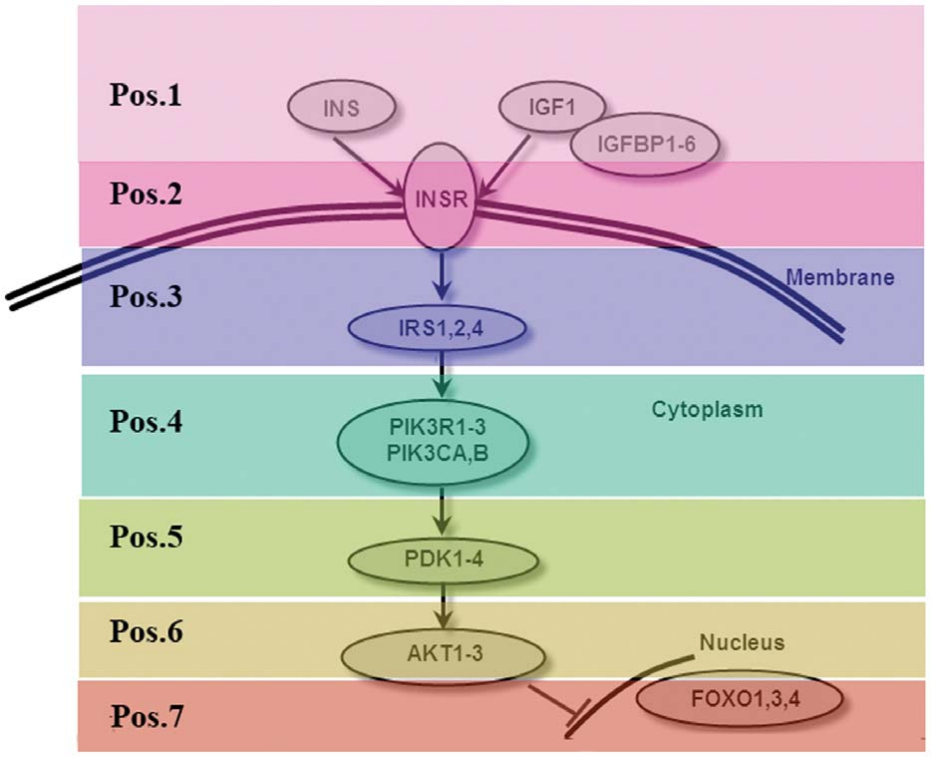
An illustration of the IGF1/FoxO pathway genes. Arrows indicate the direction of signal transduction. The numbers on the left side represent the position of the IGF1/FoxO pathway genes.

There were 28 murine genes in the IGF1/FoxO signal transduction pathway, and a simple scheme of IGF1/FoxO pathway components is shown in Figure 2 (modified from [19]). These 28 genes have been aligned to the pig chromosomes to identify orthologous genes using BLASTn program [20] from the blastall tool [21] with default parameters. We identify many pig orthologs of the murine genes and the compared results are shown in **Table 1**. However, the chromosomal locations for 4 genes (*INS2*, *IRS2*, *PIK3CA* and *PIK3CB*) are still unknown.

**Table 1:** The comparison of orthologs members between pig and murine IGF1/FoxO pathway.

Ensemble No (mouse)	Mousegenename	Mousechr.	Mouse genome position	Ensemble No (pig)	Piggenename	Pig chrby blast	Pig genome Position
**ENSMUSG00000020053**	Igf1	10	87321010–87399792	ENSSSCG00000000857	IGF1	5	77082628–77154428
**ENSMUSG00000020429**	Igfbp1	11	7097785–7102549	ENSSSCG00000016728	IGFBP1	18	48650114–48654870
**ENSMUSG00000039323**	Igfbp2	1	72871077–72899048	*	IGFBP2	15	112022157–112022652
**ENSMUSG00000020427**	Igfbp3	11	7106089–7113926	ENSSSCG00000016729	IGFBP3	18	48778107–48782267
**ENSMUSG00000017493**	Igfbp4	11	98902558–98913969	ENSSSCG00000017472	IGFBP4	12	19797073–19811336
**ENSMUSG00000026185**	Igfbp5	1	72904506–72921458	ENSSSCG00000016178	IGFBP5	15	112017411–112019845
**ENSMUSG00000023046**	Igfbp6	15	101974793–101979942	ENSSSCG00000000255	IGFBP6	5	16854964–16855290
**ENSMUSG00000000215**	Ins2	7	149864561–149885415	/	/	/	/
**ENSMUSG00000005534**	Insr	8	3150922–3279617	*	INSR	2	50637472–50720384
**ENSMUSG00000055980**	Irs1	1	82229676–82287991	ENSSSCG00000016242	IRS1	15	120872701–120981690
**ENSMUSG00000038894**	Irs2	8	10986980–11008458	/	/	/	/
**ENSMUSG00000054667**	Irs4	X	138145541–138159806	ENSSSCG00000012577	IRS4	X	88228629–88230746
**ENSMUSG00000041417**	Pik3r1	13	102450716–102538172	ENSSSCG00000016958	PIK3R1	16	44114529–44120792
**ENSMUSG00000031834**	Pik3r2	8	73292075–73300612	ENSSSCG00000013900	PIK3R2	2	62387523–62399642
**ENSMUSG00000028698**	Pik3r3	4	115894223–115975661	ENSSSCG00000003909	PIK3R3	6	118327752–118488382
**ENSMUSG00000027665**	Pik3ca	3	32296593–32367408	/	/	/	/
**ENSMUSG00000032462**	Pik3cb	9	98938821–99041040	/	/	/	/
**ENSMUSG00000006494**	Pdk1	2	71711281–71741915	ENSSSCG00000015958	PDK1	15	73606993–73615575
**ENSMUSG00000038967**	Pdk2	11	94887572–94902668	*	PDK2	12	24797209–24801353
**ENSMUSG00000035232**	Pdk3	X	91009946–91077540	ENSSSCG00000012181	PDK3	X	19401655–19471675
**ENSMUSG00000019577**	Pdk4	6	5433351–5446309	ENSSSCG00000015334	PDK4	9	70465829–70477355
**ENSMUSG00000001729**	Akt1	12	113892032–113913095	ENSSSCG00000003194	AKT1	6	38477164–38484612
**ENSMUSG00000004056**	Akt2	7	28376571–28425845	ENSSSCG00000002989	AKT2	6	33533100–33560034
**ENSMUSG00000019699**	Akt3	1	178950204–179188334	ENSSSCG00000010872	AKT3	10	16162075–16280499
**ENSMUSG00000044167**	Foxo1	3	52072259–52154031	ENSSSCG00000009370	FOXO1	11	15075341–15085340
**ENSMUSG00000048756**	Foxo3	10	41901647–41996561	ENSSSCG00000004387	FOXO3	1	78223324–78349962
**ENSMUSG00000042903**	Foxo4	X	98449867–98456212	ENSSSCG00000012399	FOXO4	X	56862227–56867783
**ENSMUSG00000052135**	Foxo6	4	119939684–119959954	ENSSSCG00000009371	FOXO6	11	15207819–15208487

Each of the genes that were mapped on the pig genome has been co-localized with published QTL from pigQTLdb. The genes identified as being co-localized with a QTL are presented in **Table S5**. FOXO1 gene has been co-localized with 22 QTL, FOXO3 gene with 48 QTL, FOXO6 gene with 32 QTL, IGFBP6 gene with 27 QTL, and PDK3 gene with 4 QTL. Only the results for the FOXO3 gene are detailed here (**Table 2**). We found that many traits were closely associated with growth and development traits, for example, average daily gain (QTL_id 5680, 329 and 5928) and feed intake (QTL_id 871). Each locus (QTL_id) corresponded to one publication.

**Table 2:** Co-localization of QTL map from Pig QTLdb and candidate gene (FOXO3) from IGF1/FoxO pathway.

QTL_ID	QTL_symbol	Trait_name	QTL_start	QTL_end
**3793**	BFT	Average backfat thickness	52423837	166525431
**5670**	BFT	Average backfat thickness	3320998	144078329
**5680**	ADG	Average daily gain (10 weeks-slaughter)	3320998	193909568
**329**	ADG	Average daily gain (25–90 kg)	32980500	109360545
**5928**	ADG	Average daily gain (on weaning)	52423837	86769928
**45**	LUMBF	Backfat at last lumbar	73990528	154961837
**3794**	LRIBF	backfat at last rib	52423837	166525431
**5674**	LRIBF	backfat at last rib	7302766	163511869
**651**	BFTR	Backfat at rump	43291013	144078329
**3795**	10RIBBFT	Backfat at tenth rib7	52423837	158592899
**5672**	10RIBBFT	Backfat at tenth rib	26500027	86769928
**5679**	BYLEAN	Belly meat content	7302766	163511869
**2845**	BELLYWT	Belly weight	51386391	139615854
**5931**	WWT	Body weight (weaning)	52423837	86769928
**4260**	CD2L	CD2-positive leukocyte number	24598042	144803085
**4261**	CD4L	CD4-positive leukocyte number	24598042	144803085
**5669**	cond	Conductivity 24 hours postmortem (ham)	52423837	166525431
**5668**	cond	Conductivity 24 hours postmortem (loin)	52423837	118433791
**6366**	CREAT	Creatinine level	33269805	126505160
**8885**	EAREA	Ear area	43291013	109360545
**8886**	EAREA	Ear area	43291013	109360545
**8853**	EARWT	Ear weight	43291013	109360545
**8854**	EARWT	Ear weight	43291013	109360545
**5678**	ECLC	Estimated carcass lean content	7302766	163511869
**5676**	FATAREA	Fat area	11402561	144078329
**5677**	FP	Fat ratio (percentage)	7302766	163511869
**871**	FEEDIN	Feed Intake	26957266	91781628
**8908**	FSCOREF	feet score (front)	32980500	108464751
**860**	HEADWT	Head weight	26957266	91781628
**5420**	HCT	hematocrit	33269805	126505160
**8910**	LSCOREH	leg score (hind)	32980500	108464751
**872**	LIVWT	Liver weight	26957266	91781628
**3796**	LMA	Loin muscle area	52423837	158592899
**5468**	LYMPH	Lymphocyte number	33269805	126505160
**2930**	MARB	Marbling	52423837	91781628
**4019**	MARB	Marbling	27710381	179573387
**78**	MARB	Marbling	73990528	86769928
**5667**	COLORO	Meat color OPTO QTL	52423837	163511869
**5664**	pH	pH for Longissmus Dorsi	52423837	118433791
**5666**	pH	pH for Longissmus Dorsi	52423837	118433791
**5665**	pH	pH for Semimembranosus	52423837	118433791
**6380**	BPOTASS	Potassium level	33269805	126505160
**9658**	PRRSVAB	PRRSV antibody titer	26500027	144251236
**5675**	SIDEF	Side fat	26500027	86769928
**5671**	SHOUFATD	Subcutaneous fat depth at shoulder	3320998	163511869
**6481**	TNUM	Teat number	43291013	109360545
**79**	TOTLIP	Total lipid	24598042	129115585
**611**	WBC	White blood cell counts	32980500	108464751

At last, 23 single nucleotide polymorphisms (SNPs) in 9 genes (*IGF1*, *IGFBP1*, *INSR*, *IRS1*, *PIK3R3*, *PDK3*, *PDK4*, *AKT3* and *FOXO3*) were identified in the IGF1/FoxO pathway, and 11 of which were predicted novel SNPs. Using this approach, 13 SNPs were determined to be polymorphic in our panel, three of which were novel SNPs (S14, S18 and S28 in **Table S3**). The allelic and genotypic frequencies, as well as the concrete distribution in this population were determined by SNaPshot method. Analysis of the same site using PCR-RFLP generated similar results to those of the SnaPshot analysis (data not shown). Our study showed that the SNaPshot method can reliably replace sequencing and RFLP testing, particularly in cases in which no restriction endonuclease recognizes the polymorphic site being analyzed. Moreover, the same results can be achieved in a shorter time by SNaPshot analysis for small or medium output.

Using both our MLM model and the likelihood ratio test, we tested the significance of the additive effect and dominance in IGF1-FoxO pathway level on each of the 5 measured traits. Our results showed that the IGF1-FoxO pathway significantly associated with BW100 (0.007) and ADG (0.010). No significant dominance effect was found in the present study.

For further identifing which SNP (or combinations of SNPs) plays a key role(s) in the IGF1-FoxO pathway, we performed single-SNP association analysis and interaction models analysis. The single SNP association analysis was initiated using linear regression with adjustment for gender and pedigree. Of the 13 SNPs analyzed, 4 SNPs (AS3, AS6, AS9 and AS10) were showing the strong association with BW100 and ADG. Overall, most of SNP associations were moderate in single SNP analysis, suggesting that there appeared to be multiple weak associations in the studied pathway. These SNPs may therefore interact with each other to exert their effect.

In order to complement pathway-based analysis and provide additional insights into the genetic architecture of studied traits, a generalized multifactor dimensionality reduction method was used to determine the relationship between interacted SNPs (combined effect) and studied traits. Several interactions were shown to be significant: a two-SNP interaction model (AS3 and S14) and a three-SNP interaction (AS6, AS9 and S18) were related BW100 as well as a two-SNP interaction (AS10 and S28) and a three-SNP interaction (AS6, AS9 and S18) involved to ADG was found. Our study indicates that the susceptibility SNPs identified by single SNP analysis might independently and/or in a combined manner influence studied trait. Overall, we can identify many SNPs of moderate effect that influence studied traits though interaction, and most of the SNPs found have been reported in the literature as functional elements correlated with the model animals’ growth [22], [23], [24].

## Discussion

Over the past decade, researchers have made great progresses in identifying economical relevance factors (genes, QTLs and SNPs) contributing to trait variation. SNPs have been the markers of choice in recent years, due to their high stability, density and the highly automated way in which SNPs are detected. Various statistical models and techniques have been developed and applied in order to make inferences about the general population using studied samples as well as relate the genotype information to observable/measurable phenotypes [25]. Our approach is different from traditional candidate gene strategies, which identify sets of SNPs/variants showing allele frequency and genotypic concordance among subjects. Parts of the traditional strategies based on the linkage disequilibrium between markers mainly consider haplotypes [26], [27], but is restricted to areas of high LD (haplotype blocks). In addition, clustering and principal components were also used to group markers based on LD [28], [29]. Unlike these, our CGSA approach can be considered as an improvement of traditional candidate gene approach. We group markers based on functional considerations, which we believe is in better concordance with the goal of identifying variation that is associated with phenotype.

In order to improved statistical power, several similar candidate pathway approaches [30], [31] were published to deal with some of the same issues as our study. However, our study was conducted differently in a variety of ways. First, our method can make full use of the large scale study results performed on model animals (human, mouse) which have provided the potential to define candidate gene sets to identify the complex processes involved in the phenotype. Second, our approach takes full advantage of previously reported QTL, candidate gene sets that lie within the region of the QTL, and that have physiological relevance to the trait (considered as primary candidates for the QTL), particularly within multiple overlapping QTL of interest. Third, most of existing candidate pathway approaches [30] use multiple regression models to test the significance, but our method allows us to test whether a set of genes are associated with a trait of interest based on MLM framework which could eliminate false positives effectively both in candidate gene association study and GWAS [32], [33].

Using our approach, we succeed in identifying combined effect members in our predefined set, which is associated with several economical traits in pigs. The increase in power when compared to single-marker approaches based on the joint reduction of false positives and false negatives. Reducing false negatives comes from the fact that moderately strong signals can be combined into a stronger signal, whereas, these members might be ignored by strong global significance in single SNP analysis. Reducing false positives is because of our method based on MLM framework which could eliminate false positives effectively [32], [33]. In the present case study, 2 SNPs associated with the BW 30, 1 SNP with BW 60 in single-marker approach were excluded in case of false positives because our gene set approach didn’t indicate the significant association in the pathway level. In the present case study, no significant dominance effect was found because sample size is relatively too small. So, the real results cannot demonstrate the advantage of the new approach. Our group is trying to apply our approach to other dataset with large sample size to verify the relevant conclusions furtherly.

Finally, the results from this approach offer a clearer picture on the functional relevance of the discoveries. The significant SNPs found using this approach warrant further experimental exploration. Especially, SNPs from FoxO3, which were not only significant in single SNP analysis, but also in combined effect analysis. This FoxO3 gene was overlapped with multiple QTLs (48 QTLs) of pigs’ growth and development traits, and was reported to be related to muscle growth abundantly [34], [35], [36]. The validation experiments would be more or less complex depending on the nature of the trait (mono- or polygenic). As biotechnology and genetic transformation techniques advance, we will be able to demonstrate experimentally whether specific SNPs truly determine variation in a particular trait.

It has become clear that gene sets rather than individual gene are essential in understanding carcinoma [37], [38]. One drawback of this approach, however, is the reliance of prior assumptions that genes identified in the QTL regions are well annotated, the explicitly recording from KEGG and so on. If not, we may overlook or miss some genes or SNPs involved in the phenotype for incompleteness of annotation and complex biological process.

### Conclusions

We have proposed a novel approach for determining specific genes and their SNPs that are associated with studied traits in the gene set level. The simulation results demonstrated that our CGSA performed higher statistical power over the traditional candidate gene approach. We also used this approach to study the relationship between IGF1-FoxO pathway and growth and development traits of pig. The results suggested that genetic variations in the IGF1-FoxO pathway modulate the growth and development in pigs. The CGSA is flexible to be extended to model complex genetic structures and can be applied by other gene sets. Computer programs (R and perl source code) are available at http://klab.sjtu.edu.cn/CGSA.

## Supporting Information

Table S1 **The 57 SNPs with their positions in the insulin and insulin growth factor-1/FoxO (IGF1-FoxO).**
(XLS)

Table S2 **The main effects and the effects of interaction (epistatic effects) of the makers from the IGF1/FoxO signal transduction pathway in the simulation experiment.**
(XLS)

Table S3 **The primer sequences for analysis of 23 identified SNPs.**
(XLS)

Table S4 **The comparment of statistical power between our CGSA and candidate gene approach.**
(XLSX)

Table S5 **The genes from the IGF1/FoxO signal transduction pathway identified as being co-localized with published QTL from pigQTLdb.**
(XLS)

## References

[1] Rothschild M, Soller M (1997) Candidate gene analysis to detect genes controlling traits of economic importance in domestic livestock. Probe 8: 13–20.

[2] Short TH, Rothschild MF, Southwood OI, McLaren DG, de Vries A, et al (1997) Effect of the estrogen receptor locus on reproduction and production traits in four commercial pig lines. J Anim Sci 75: 3138–3142. 9419986 10.2527/1997.75123138x

[3] Hu ZL, Fritz ER, Reecy JM (2007) AnimalQTLdb: a livestock QTL database tool set for positional QTL information mining and beyond. Nucleic Acids Res 35: D604–609. 17135205 10.1093/nar/gkl946 PMC1781224

[4] Ashburner M, Ball CA, Blake JA, Botstein D, Butler H, et al (2000) Gene ontology: tool for the unification of biology. The Gene Ontology Consortium. Nat Genet 25: 25–29. 10802651 10.1038/75556 PMC3037419

[5] Kanehisa M, Goto S (2000) KEGG: kyoto encyclopedia of genes and genomes. Nucleic Acids Res 28: 27–30. 10592173 10.1093/nar/28.1.27 PMC102409

[6] Subramanian A, Tamayo P, Mootha VK, Mukherjee S, Ebert BL, et al (2005) Gene set enrichment analysis: a knowledge-based approach for interpreting genome-wide expression profiles. Proc Natl Acad Sci U S A 102: 15545–15550. 16199517 10.1073/pnas.0506580102 PMC1239896

[7] Wang Q, Wamg M, Yang Y, Pan Y (2011) SNPpath: Characterizing cattle SNPs by enriched pathway terms. Animal Science Journal 83: 279–283. 22515686 10.1111/j.1740-0929.2011.00952.x

[8] Ron M, Israeli G, Seroussi E, Weller JI, Gregg JP, et al (2007) Combining mouse mammary gland gene expression and comparative mapping for the identification of candidate genes for QTL of milk production traits in cattle. BMC Genomics 8: 183. 17584498 10.1186/1471-2164-8-183 PMC1906769

[9] Myers EW, Miller W (1988) Optimal alignments in linear space. Comput Appl Biosci 4: 11–17. 3382986 10.1093/bioinformatics/4.1.11

[10] Wernersson R, Schierup MH, Jorgensen FG, Gorodkin J, Panitz F, et al (2005) Pigs in sequence space: a 0.66X coverage pig genome survey based on shotgun sequencing. BMC Genomics 6: 70. 15885146 10.1186/1471-2164-6-70 PMC1142312

[11] Purcell S, Neale B, Todd-Brown K, Thomas L, Ferreira MAR, et al (2007) PLINK: a tool set for whole-genome association and population-based linkage analyses. The American Journal of Human Genetics 81: 559–575. 17701901 10.1086/519795 PMC1950838

[12] Henderson C (1985) Best linear unbiased prediction of nonadditive genetic merits in noninbred populations. Journal of animal science 60: 111–117.

[13] Calle ML, Urrea V, Malats N, Van Steen K (2010) mbmdr: an R package for exploring gene-gene interactions associated with binary or quantitative traits. Bioinformatics 26: 2198–2199. 20595460 10.1093/bioinformatics/btq352

[14] Mahachie John JM, Van Lishout F, Van Steen K (2011) Model-Based Multifactor Dimensionality Reduction to detect epistasis for quantitative traits in the presence of error-free and noisy data. Eur J Hum Genet 19: 696–703. 21407267 10.1038/ejhg.2011.17 PMC3110049

[15] Fan B, Onteru SK, Du ZQ, Garrick DJ, Stalder KJ, et al (2011) Genome-wide association study identifies loci for body composition and structural soundness traits in pigs. PloS one 6: e14726. 21383979 10.1371/journal.pone.0014726 PMC3044704

[16] Lipka AE, Tian F, Wang Q, Peiffer J, Li M, et al (2012) GAPIT: genome association and prediction integrated tool. Bioinformatics 28: 2397–2399. 22796960 10.1093/bioinformatics/bts444

[17] Wang M, Wang Q, Zhang X, Yang Y, Zhao H, et al (2011) Uncovering the transcriptional circuitry in skeletal muscle regeneration. Mamm Genome 22: 272–281. 21509518 10.1007/s00335-011-9322-x

[18] Gilmour A, Gogel B, Cullis B, Thompson R, Butler D, et al. (2009) ASReml user guide release 3.0. VSNi website.Available: http://www.vsni.co.uk/software/asreml. Accessed 2012 Apr 3.

[19] Taniguchi CM, Emanuelli B, Kahn CR (2006) Critical nodes in signalling pathways: insights into insulin action. Nature Reviews Molecular Cell Biology 7: 85–96. 16493415 10.1038/nrm1837

[20] Altschul SF, Gish W, Miller W, Myers EW, Lipman DJ (1990) Basic local alignment search tool. J Mol Biol 215: 403–410. 2231712 10.1016/S0022-2836(05)80360-2

[21] Altschul SF, Madden TL, Schaffer AA, Zhang J, Zhang Z, et al (1997) Gapped BLAST and PSI-BLAST: a new generation of protein database search programs. Nucleic Acids Res 25: 3389–3402. 9254694 10.1093/nar/25.17.3389 PMC146917

[22] Soroceanu L, Kharbanda S, Chen R, Soriano RH, Aldape K, et al (2007) Identification of IGF2 signaling through phosphoinositide-3-kinase regulatory subunit 3 as a growth-promoting axis in glioblastoma. Proceedings of the National Academy of Sciences 104: 3466–3471. 10.1073/pnas.0611271104 PMC1802005 17360667

[23] Schiaffino S, Mammucari C (2011) Regulation of skeletal muscle growth by the IGF1-Akt/PKB pathway: insights from genetic models. Skelet Muscle 1: 4. 21798082 10.1186/2044-5040-1-4 PMC3143906

[24] Williamson DL, Raue U, Slivka DR, Trappe S (2010) Resistance exercise, skeletal muscle FOXO3A, and 85-year-old women. J Gerontol A Biol Sci Med Sci 65: 335–343. 20139145 10.1093/gerona/glq005 PMC2844061

[25] Liang Y, Kelemen A (2006) Associating phenotypes with molecular events: recent statistical advances and challenges underpinning microarray experiments. Funct Integr Genomics 6: 1–13. 16292543 10.1007/s10142-005-0006-z

[26] Ginja C, Telo da Gama L, Penedo MC (2009) Y chromosome haplotype analysis in Portuguese cattle breeds using SNPs and STRs. J Hered 100: 148–157. 18832111 10.1093/jhered/esn080

[27] Meidtner K, Schwarzenbacher H, Scharfe M, Severitt S, Blocker H, et al (2009) Haplotypes of the porcine peroxisome proliferator-activated receptor delta gene are associated with backfat thickness. BMC Genet 10: 76. 19943979 10.1186/1471-2156-10-76 PMC3087513

[28] Durrant C, Zondervan KT, Cardon LR, Hunt S, Deloukas P, et al (2004) Linkage disequilibrium mapping via cladistic analysis of single-nucleotide polymorphism haplotypes. Am J Hum Genet 75: 35–43. 15148658 10.1086/422174 PMC1182006

[29] Gauderman WJ, Murcray C, Gilliland F, Conti DV (2007) Testing association between disease and multiple SNPs in a candidate gene. Genetic epidemiology 31: 383–395. 17410554 10.1002/gepi.20219

[30] Lesnick TG, Papapetropoulos S, Mash DC, Ffrench-Mullen J, Shehadeh L, et al (2007) A genomic pathway approach to a complex disease: axon guidance and Parkinson disease. PLoS genetics 3: e98. 17571925 10.1371/journal.pgen.0030098 PMC1904362

[31] García-Gámez E, Reverter A, Whan V, McWilliam SM, Arranz JJ, et al (2011) Using Regulatory and Epistatic Networks to Extend the Findings of a Genome Scan: Identifying the Gene Drivers of Pigmentation in Merino Sheep. PloS one 6: e21158. 21701676 10.1371/journal.pone.0021158 PMC3119053

[32] Zhang Z, Ersoz E, Lai CQ, Todhunter RJ, Tiwari HK, et al (2010) Mixed linear model approach adapted for genome-wide association studies. Nature genetics 42: 355–360. 20208535 10.1038/ng.546 PMC2931336

[33] Yu J, Pressoir G, Briggs WH, Bi IV, Yamasaki M, et al (2005) A unified mixed-model method for association mapping that accounts for multiple levels of relatedness. Nature genetics 38: 203–208. 16380716 10.1038/ng1702

[34] Hribal ML, Nakae J, Kitamura T, Shutter JR, Accili D (2003) Regulation of insulin-like growth factor-dependent myoblast differentiation by Foxo forkhead transcription factors. J Cell Biol 162: 535–541. 12925703 10.1083/jcb.200212107 PMC2173790

[35] Mammucari C, Milan G, Romanello V, Masiero E, Rudolf R, et al (2007) FoxO3 controls autophagy in skeletal muscle in vivo. Cell Metab 6: 458–471. 18054315 10.1016/j.cmet.2007.11.001

[36] Dentice M, Marsili A, Ambrosio R, Guardiola O, Sibilio A, et al (2010) The FoxO3/type 2 deiodinase pathway is required for normal mouse myogenesis and muscle regeneration. The Journal of clinical investigation 120: 4021–4030. 20978344 10.1172/JCI43670 PMC2964991

[37] Wood LD, Parsons DW, Jones S, Lin J, Sjo blom T, et al (2007) The genomic landscapes of human breast and colorectal cancers. Science 318: 1108–1113. 17932254 10.1126/science.1145720

[38] Vogelstein B, Kinzler KW (2004) Cancer genes and the pathways they control. Nature medicine 10: 789–799. 10.1038/nm1087 15286780

